# Skeletal muscle microvascular and respiratory responses to exercise in healthy and T2D adults: agreement and reproducibility study of NIRS technologies

**DOI:** 10.1007/s00421-026-06300-y

**Published:** 2026-06-29

**Authors:** Adam A. Luco, Martin Gram, Inge Ramakers, Terence Ryan, Osvaldo Enriquez, James Faulkner, Lee Stoner, David S. Rowlands

**Affiliations:** 1 School of Sport, Exercise and Nutrition, Massey University, Albany Expressway (SH17), Albany, Auckland, 0632 New Zealand; 2 College of Health and Human Performance, University of Florida, Florida, USA; 3 Automation Controls, Applied Medical, Rancho Santa Margarita, CA USA; 4 Primary Care Research Centre, University of Southhampton, Southhampton, UK; 5 Scienceye, Hillsborough, NC USA

**Keywords:** Microvascular perfusion, Microvascular blood flow, Isokinetic, Haemoglobin, Near-infrared spectroscopy

## Abstract

**Introduction:**

Continuous wave (CW) near-infrared spectroscopy (NIRS) is widely used in skeletal muscle tissue hemodynamic and respiratory research utilizing fixed light scattering assumptions.

**Purpose:**

Compare reproducibility and technical characteristics of CW-NIRS against frequency-domain (FD)-NIRS, which allows for intra-individual scattering correction.

**Methods:**

Muscle blood flow (mBF) and oxygen uptake (${\mathrm{m}\dot{\mathrm{V}}\mathrm{O}}_{2}$) (using rapid-occlusions), and perfusion were assessed with NIRS in 10 healthy (ND) and 10 type-2 diabetic (T2D) men at rest and during knee extensions at 5% and 15% of maximal voluntary contraction (MVC) on three occasions within 10 days.

**Results:**

In both groups, exercise responses for mBF and ${\mathrm{m}\dot{\mathrm{V}}\mathrm{O}}_{2}$ were not substantially different between technology with moderate-high between-day reproducibility (ICC range 0.72–0.98). However, compared to FD-NIRS, CW-NIRS perfusion substantially higher at rest (21 uM; 90%CI 29–13), 5%MVC (18 uM; 27–9) and 15%MVC (17 uM; 26–8) in T2D, but not ND. Standardized typical errors during exercise were small-moderate: e.g., 15%MVC mBF, CW-NIRS (ND 0.45; T2D 0.49), FD-NIRS (0.35; 0.49); mV̇O_2_, CW-NIRS (0.24; 0.26), FD-NIRS (0.45; 0.32); perfusion CW-NIRS (0.25; 0.25) and FD-NIRS (0.25; 0.23); and, not different between groups and technology.

**Conclusion:**

Both NIRS technologies are reproducible. CW-NIRS assumptions work for muscle blood flow and oxygen use at rest and during exercise. FD-NIRS might be better at detecting changes in perfusion during exercise, especially in older or clinical groups. However, FD-NIRS is more expensive and complex, making it less practical.

**Supplementary Information:**

The online version contains supplementary material available at https://doi.org/10.1007/s00421-026-06300-y.

## Introduction

Near-infrared spectroscopy (NIRS) is a non-invasive optical technique used to assess skeletal muscle hemodynamics and oxygen transfer by measuring oxygenated ([O₂Hb]), deoxygenated ([HHb]), and total hemoglobin ([tHb]) concentrations (Jue and Masuda 2013; Stone et al. 2016). When combined with blood-flow occlusion and exercise protocols, NIRS can estimate muscle blood flow (mBF), oxygen consumption (${\mathrm{m}\dot{\mathrm{V}}\mathrm{O}}_{2}$), and tissue perfusion (Van Beekvelt et al. 2001a, b). These capabilities make NIRS a valuable tool for studying skeletal muscle microvascular function and metabolism, particularly in clinical conditions characterized by impaired oxygen delivery and utilization, such as type 2 diabetes (T2D) (Scholkmann et al. 2014). Accordingly, NIRS has been widely applied to assess microvascular function, estimate mitochondrial oxidative capacity, and evaluate responses to exercise interventions.

Despite its growing use, interpretation of NIRS signals is complicated by the highly scattering and heterogeneous nature of biological tissue. Photons traveling through tissue follow complex paths rather than straight trajectories, meaning detected light intensity is influenced by both absorption (μa) and scattering (μs). Because hemoglobin quantification depends on these optical properties, differences in how NIRS technologies address absorption and scattering can influence measurement accuracy (Grassi and Quaresima 2016).

Continuous-wave (CW) NIRS, the most commonly used approach in skeletal muscle research, measures changes in light attenuation using the modified Beer–Lambert law and assumes constant optical properties. While this method enables portable and high-temporal-resolution measurements suitable for exercise studies, it cannot independently quantify absorption and scattering and therefore relies on literature-derived correction factors such as the differential pathlength factor (DPF) (Hammer et al. 2019). These constants are typically derived from limited tissue sites and applied uniformly across individuals (Duncan et al. 1995), despite evidence that scattering varies across muscles, between populations, and during dynamic exercise. Such variability may introduce error in both absolute and relative hemoglobin measurements.

In contrast, time-domain (TD) and frequency-domain (FD) NIRS quantify both absorption and scattering by measuring the temporal or phase characteristics of detected light. This allows estimation of true photon pathlength and enables calculation of absolute hemoglobin concentrations (Fantini and Sassaroli 2020). Direct quantification of scattering may be particularly important in populations with obesity or T2D, where increased adiposity, fibrosis, and altered perfusion can further influence tissue optical properties. Exercise may also modify scattering through tissue compression and metabolic shifts, potentially affecting NIRS-derived measures.

We previously reported acceptable reproducibility of CW-NIRS combined with rapid venous occlusions (VO) and arterial occlusions (AO) for assessing leg mBF and ${\mathrm{m}\dot{\mathrm{V}}\mathrm{O}}_{2}$ responses, respectively, to exercise in healthy individuals (Lucero et al. 2018). However, the extent to which assumptions regarding constant scattering influence these outcomes remains unclear. Moreover, the validity and reproducibility of skeletal muscle NIRS measurements in metabolically diseased muscle, such as in T2D, remain insufficiently characterized.

Therefore, the purpose of this study was to evaluate the quantitative agreement and reproducibility of hemodynamic and respiratory measurements obtained using CW-NIRS and FD-NIRS in healthy non-diabetic (ND) and T2D individuals at rest and during dynamic exercise. Because FD-NIRS directly quantifies scattering whereas CW-NIRS assumes constant optical properties, we hypothesized that CW-NIRS would produce systematically offset estimates of [tHb] compared with FD-NIRS. We further hypothesized that CW-NIRS measurements would demonstrate greater variability and lower precision, reflected by larger standardized typical error and wider confidence intervals, reducing sensitivity to detect device- and group-related differences.

## Methods

### Participants

Ten young, healthy, physically active men (defined as meeting the daily exercise guidelines according to the American College of Sports Medicine (Albright et al. 2000)) and 10 sedentary non-insulin dependent men with T2D (HbA1c ≥ 48 mmol/L, diagnosed minimum 1 y) were recruited into the study (Table 1). All participants were of Caucasian ancestry and skin tone was not recorded. As the experiment was designed to establish the reproducibility of the NIRS devices in ND and T2D populations, the participants were not age-matched between groups. All participants were excluded if they were a current smoker or smoked 6 months prior, used β-blockers, injured, had lower limb surgery in the past 4 weeks, moderate to severe retinopathy, nephropathy or neuropathy, history of cerebrovascular or cardiovascular diseases. Due to NIRS measurement limitations, participants were also excluded if they had greater than 2 cm of adipose tissue thickness (ATT) over the NIRS probe region of interest as measured by ultrasound (Van Beekvelt et al. 2001a, b)⁠. Ethical approval was obtained from the institutional Human Ethics Committee, and all participants providing written informed consent.

**Table 1:** Mean values and standard deviations for participant characteristics

Group	Statistic	Age	Height (m)	Weight (kg)	ATT (cm)	VL (cm)	VL Belly (cm)
T2D	Mean	56.3	173.6	91.7	0.93	2.17	3.71
	SD	5.1	6.9	14.4	0.43	4.20	1.39
ND	Mean	22.5	173.2	68.8	0.44	3.14	1.95
	SD	3.40	6.38	10.6	0.14	4.00	0.26

Abbreviations: *ATT* adipose tissue thickness, *VL* vastus lateralis, *VL Belly* distance from skin to the belly of the VL calculated as ATT + 1/2*VL

### Experimental design and familiarisation

**Fig. 1:**
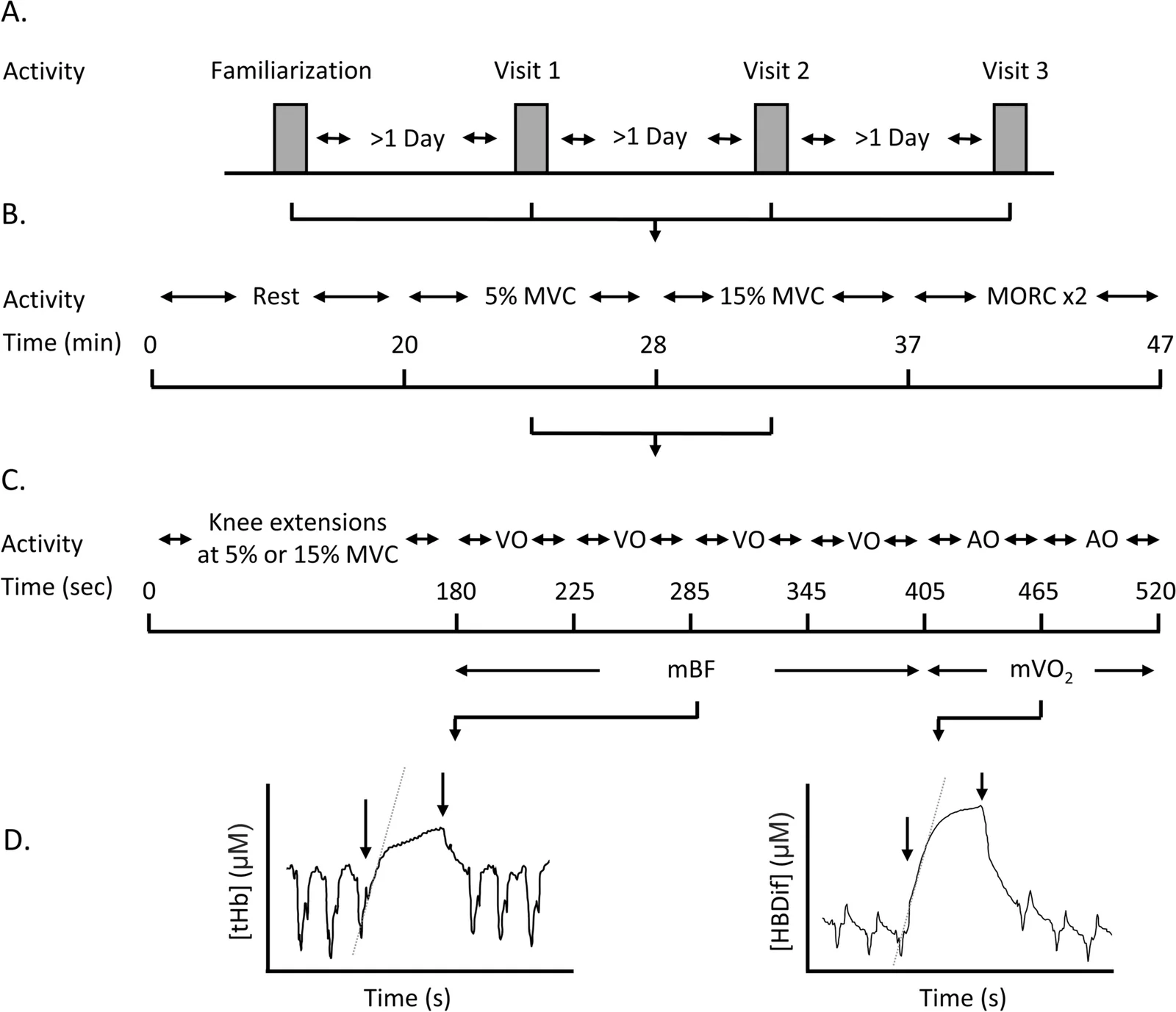
Experimental overview. **A** Each participant completed a total of 4 laboratory visits, one familiarization session and 3 test days allowing for at least 1 day in between tests. **B** Timeline of a single visit highlighting tests done. After experimental set-up, participant rested for 20 min to obtain resting or baseline measures, then completed 3 min of knee extension exercise at 5 then 15% MVC before exercise measures were taken. Following, participants did a 15 s bout of knee extension exercise before a series of AOs for MORC test which was repeated twice. **C** Timeline of a single exercise bout showing 3 min of knee extension before measures. Exercising leg is suspended in a relaxed position before the VO or AO inflates for 10 s, then the exercise is resumed for 45 s before the next measure. **D** Example NIRS traces of the NIRS signal with arrows showing the occlusion inflation and deflation. Gray dashed line shows the linear regression used to calculate mBF (via VO) and ${\mathrm{m}\dot{\mathrm{V}}\mathrm{O}}_{2}$ (via AO). On the left the tHb signal during last three knee extensions followed by VO then continuation of exercise. On the right the ½ * △[HbDif] signal is used to calculate the subsequent change in oxygenation in the occluded muscle. Abbreviations. *AO* arterial occlusion, *MVC* maximal voluntary contraction, *mBF* muscle blood flow, ${\mathrm{m}\dot{\mathrm{V}}\mathrm{O}}_{2}$ muscle oxygen consumption, *MORC* muscle oxygen recovery capacity

This study utilized a repeated-measures design to assess the reproducibility of NIRS-derived mBF and ${\mathrm{m}\dot{\mathrm{V}}\mathrm{O}}_{2}$. Following a familiarization trial, participants completed three identical experimental visits on non-consecutive days within a 10-day period (Fig. 1). All testing occurred in a temperature-controlled environment (20.5 °C, SD 0.8).

During familiarization, participants were accustomed to the AO and VO protocols and performed a maximum voluntary contraction (MVC) test on an isokinetic dynamometer (Biodex Medical Systems, Inc. Shirly, NY, USA), by way of repetitions of several trials where the participant rehearsed the positioning and relaxation of the leg during the occlusions. To optimize experimental measures, participants were seated in a 70° reclined seat to achieve a 110° hip angle and a 90° knee extension angle (Grassi and Quaresima 2016). The settings were adjusted so that the axial portion of the knee aligned with the axis of rotation on the dynamometer to maximize VL activation. Participants were instructed and given verbal encouragement to kick as hard as possible for 5 s and the output force, maximum voluntary contraction (MVC), was recorded as the peak value of three trials with 5 min rest in between trials. This max force was used to determine 5% and 15% individual exercise workloads_._

### Near-Infrared Spectroscopy (NIRS) and Instrumentation

Two NIRS devices were used to continuously monitor changes in local concentrations of [O_2_Hb], [HHb], and [tHb]. One device was placed on the VL of the dominant leg and the other was oppositely placed on the VL of the non-dominant leg. The NIRS devices were randomized between legs. The CW-NIRS device (PortaLite; Artinis Medical Systems BV, Elst, The Netherlands) consists of six light-emitting diodes (760 and 850 nm) from 3 transmission distances of 3.0, 3.5, and 4.0 cm from the detector. A DPF of 4.0, h of 0.00046 nm^−1^, and k of 1.1 mm^−1^ were used to correct for scattering and absorption, and data were collected at 10 Hz. The CW-NIRS hemoglobin concentration values were calculated from transmitter 2 allowing for a theoretical penetration distance of 1.75 cm. The FD-NIRS device (Oxiplex TS, ISS Inc., IL) consists of eight light-emitting (690 and 830 nm) diodes from four transmission distances of 2.5, 3.0, 3.5, 4.0 cm from the detector. Unlike the CW-NIRS, all four transmitters on the FD-NIRS are used to determine a best-fit slope of the amplitude of modulation (AC or DC), and phase shift (time of flight) components versus transmitter-detector distance used to continuously determine the degree of scattering, so it can be accurately accounted for to determine hemoglobin concentrations (Fantini et al. 1994)⁠. The instrument was calibrated before each measurement by positioning the probe on calibration blocks with known scattering and absorption coefficients. The FD-NIRS-calculated hemoglobin concentrations were smoothed using a moving average window of 0.5 s.

For both NIRS devices, following SENIAM guidelines (Hermens et al. 2000), the probe was securely adhered using double-sided tape to the skin parallel to the muscle fibers, about two-thirds on the line from the anterior spina ilica superior to the lateral side of the patella, over the VL muscle belly using a custom-built guide and verified using ultrasound. A custom-made cover shielded the probe from ambient light while allowing it to move with the skin during contractions minimizing changes in contact pressure (Hamaoka et al. 2011). The probe position was marked with a pen to ensure that the same position was used for all three trials. The thickness of the VL at the probe location, along with adipose tissue thickness, were determined using B-mode ultrasound (Terason, United Medical Instruments Inc., San Jose, CA).

### Experimental protocols, data processing and parameter calculation

Experimental visits were conducted between 0700 and 1000 h following an overnight fast. Participants refrained from caffeine, alcohol, and strenuous activity for 24 h prior to testing. At each of the three visits the NIRS parameters mBF, ${\mathrm{m}\dot{\mathrm{V}}\mathrm{O}}_{2}$, [tHb] as an index of perfusion, tissue saturation, contraction-relaxation perfusion change, and the ${\mathrm{m}\dot{\mathrm{V}}\mathrm{O}}_{2}$ recovery rate constant (k) as an index of muscle oxygen recovery capacity (MORC) were measured using protocols described elsewhere (Lucero et al. 2018; Ryan et al. 2012)⁠ and summarized in Fig. 1. A 90-cm rapid-inflation cuff (~ 0.5 s; Hokanson SC 10D, DE Hokanson, Inc., Bellevue, WA) was placed as high as possible around the proximal thigh, minimizing patient discomfort and avoiding artifact motion in the NIRS signal. This cuff was connected to a custom-made rapid-inflation device that inflated the cuff to a specific pressure in less than 0.5 s (Lucero et al. 2018)⁠. Occlusions for mBF and mV̇O_2_ estimations occurred in the active leg during brief pauses where the leg was suspended in a 150º knee-joint angle permitting a relaxed muscle length, thereby facilitating blood flow (Miura et al. 2004) (Fig. 1). The non-working leg was suspended in a 150º knee-joint angle throughout.

Following 20 min of quiet rest, baseline measurements of mBF and mV̇O_2_ were assessed from the average of 4 VO and 2 AO, respectively. Each occlusion was separated by 45 s of rest with occlusion durations of 15 s and 30 s for VO and AO, respectively (Southern et al. 2014). The participant then completed 2 stages of progressive intensity (5 and 15% MVC) rhythmic isotonic knee extension exercise (1 extension/4 s) on the dynamometer (Watanabe and Akima 2011) (Fig. 1). This method was used to maximize VL contribution and standardize force output from the participant, as once the % MVC was reached the leg was allowed to freely swing to the elevated position. These intensities were reproducible without inducing fatigue (Lucero et al. 2018). Participants were instructed to contract to full extension and then allow their leg to fall back to the starting position. The participant exercised continuously for 3 min prior to the first occlusion to increase chances of achieving steady state in hemodynamic parameters and oxygen uptake. Immediately after the contraction phase of the last knee extension for each measurement point, as the leg was falling, the dynamometer was locked to hold the leg in the 150º knee-joint angle, and the cuff was inflated for 10 s. Exercise was then resumed for 45 s to maintain steady state before another measurement was collected (see below for specific procedural details for each measurement). Complete occlusion during AO at rest and exercise was verified with Doppler ultrasound in the femoral artery during pilot trials and confirmed during testing by the cessation of the pulsatile motion in the tHb signal. Following the exercise stages, the participant completed two MORC assessments, which consisted of a brief 15-s bout of light isotonic exercise (16 N·m) followed by 7 min of resting with the active leg having a 150º knee-joint angle during repeated AOs. The entire protocol was then repeated in the opposite leg using the other NIRS device. The starting leg and NIRS device were randomized for the first visit and repeated for the remaining two visits.

### Specific procedures, data processing and parameter calculation

#### Local skeletal-muscle blood flow

Estimates of mBF were obtained from the [∆tHb] signal during VO with analyzed by linear regression (Cross and Sabapathy 2017; van Beekvelt et al. 2001a, b). The rapid-inflation cuff tourniquet was inflated to a sub diastolic pressure (60–80 mmHg) to occlude venous outflow without impeding arterial inflow, thus causing venous volume to increase at a rate proportional to arterial inflow. For both NIRS devices, the slope of the [tHb] signal during the first cardiac cycle immediately after VO was defined using the pulsatile motion of the [tHb] signal. To determine mBF, the slope of the tHb signal over one cardiac cycle was obtained for 4 VOs, averaged, and converted into mL·min^−1^·100 mL^−1^, where mL is blood volume (Table 2).

**Table 2:** Equations and parameters for skeletal muscle blood flow and oxygenation, and statistical parameters for estimates of reliability

Equation (Reference)	Description	Units	Parameters and description			
			1	2	3	4
mBF = [∆tHb]/∆t · 1/C (van Beekvelt et al. 2001a, b)	Skeletal muscle blood flow (mBF) from the average rate of tHb increase under VO_2_ accounting for the molecular mass of hemoglobin (64.458 g∙mol^−1^) and the hemoglobin:O_2_ molecular ratio (1:4)	mL·min^−1^·100 mL^−1^	[∆tHb]/∆t Rate of blood ∆[tHb] (slope) during the first cardiac cycle under VO	C Blood [tHb]; assumed values for female and male participants of 7.5 and 8.5 mmol∙L^−1^, respectively	N/A	N/A
${\mathrm{m}\dot{\mathrm{V}}\mathrm{O}}_{2}$ = abs[([∆HbDif/∆t * ½] ∙ 60)/ (10 ·1.04) ·4] ∙ 22.4/1000 (van Beekvelt et al. 2001a, b)	Skeletal muscle oxygenation (${\mathrm{m}\dot{\mathrm{V}}\mathrm{O}}_{2}$) derived from the average change in HbDif during AO assuming 22.4 L for the volume of gas (STPD) and 1.04 kg∙L^−1^ for muscle density	O_2_(mL)∙min^−1^∙100 g^−1^, where g is grams of tissue	∆HbDif/∆t Rate of change in HbDif (slope) under AO. Taking the Hb difference improves signal to noise ratio but doubles the rate; multiplying by 1/2 corrects	N/A	N/A	N/A
y(t) = end – Δ ∙ e^−kt^ (Ryan et al. 2012)	Muscle oxygen recovery capacity fit to a mono-exponential curve	mLO_2_∙min^−1^∙100 g^−1^	end ${\mathrm{m}\dot{\mathrm{V}}\mathrm{O}}_{2}$ immediately after the cessation of exercise	Δ change in mV̇O_2_ from rest to exercise	k fitting rate constant	t time
TE = ∆SD/√2 (Hopkins et al. 2009)	Typical error (TE) as standard error of measurement and before standardization	Unit of measure	∆SD standard deviation of the change score	N/A	N/A	N/A
STE = TE/SD ∙ c (Hopkins et al. 2009)	Standardized TE	Unitless	SD the basal SD in the same units as TE for the appropriate reference group and/or device	c small sample-size adjustment (1-(3/4df-1), where df is degrees of freedom n-1) according to Hedges (1981)		
CV = 100*SD-100 (Hopkins et al. 2009)	Coefficient of Variation (CV) is the SD expressed as a percentage	%				
ICC = 1-TE2/SDb_2_ (Hopkins et al. 2009)	Test to test reliability	ICC Intraclass correlation coefficient	TE typical error	Sdb mean between-participant standard deviation	N/A	N/A

Additional abbreviations: *AO* arterial occlusion, *N/A* not applicable, *tHb* total hemoglobin, *VO* venous occlusion, *HbDif* hemoglobin difference ([∆HbDif] = [∆HbO_2_] – [∆HHb])

#### Skeletal muscle oxygen consumption

Estimates of ${\mathrm{m}\dot{\mathrm{V}}\mathrm{O}}_{2}$ from both NIRS devices were calculated as 1/2 * the rate of change in the hemoglobin difference signal ([∆HbDif] = [∆HbO_2_] – [∆HHb]) during arterial occlusion, analyzed using linear regression (Ryan et al. 2012)⁠. Briefly, the tourniquet was rapidly (~ 0.5 s) inflated to a supra-systolic pressure (250–300 mmHg) to occlude both venous outflow and arterial inflow, completely arresting blood flow, resulting in an increase of [HHb] and simultaneous decrease in [HbO_2_] as oxygen is released from hemoglobin and consumed by the surrounding muscle tissue (van Beekvelt et al. 2001a, b)⁠. After correcting for perfusion changes (Ryan et al. 2012)⁠, the slope of the [HbDif] signal was converted into mL O_2_·min^−1^·100 g^−1^ tissue (Table 2). With both mBF and ${\mathrm{m}\dot{\mathrm{V}}\mathrm{O}}_{2}$ the ratio can be calculated (mBF/${\mathrm{m}\dot{\mathrm{V}}\mathrm{O}}_{2}$) showing the relationship of O_2_ consumption and delivery.

#### Skeletal muscle perfusion index and tissue saturation of oxygen

Local skeletal muscle perfusion index ([tHb]) and tissue saturation were calculated as the relative average perfusion ([tHb] signal) and tissue saturation, respectively, for a given period. The [tHb] signal has been said to reflect microvascular perfusion (Ijichi et al. 2005), an indication of local O_2_ diffusing capacity (Groebe and Thews 1990). Given that the differing NIRS technologies may produce different absolute [tHb], a relative or normalized perfusion (N-[tHb]) measure was calculated as the ∆[tHb] from rest to a given exercise intensity. Tissue saturation represents the saturation of oxygen in the blood given as a percentage, calculated as [O_2_Hb]/([O_2_Hb] + [HHb]) * 100 using absolute values of [O_2_Hb] and [HHb]. Due to the limitations of CW-NIRS, SaO_2_ must be calculated from estimated measures of absolute O_2_Hb and HHb using principles of spatially resolved spectroscopy (Suzuki 1999) and is referred to as the TSI%. After resting measurements of mBF and ${\mathrm{m}\dot{\mathrm{V}}\mathrm{O}}_{2}$ were taken, the participant’s leg was lowered to 90˚ knee-joint angle in preparation for the exercise protocol. The participant then continued to rest to allow the [tHb] and tissue saturation signals to stabilize for 30 s, which was used for the resting perfusion. During exercise, the average signal values during 2-s rest period (when the leg was positioned at 90˚) between the last 8 extensions (last 30 s of exercise before occlusions) were averaged.

#### Skeletal muscle perfusion during exercise

Exercise induced perfusion change was the average change in the [tHb] across the last 8 contraction-relaxation cycles of knee-extension exercise. To our understanding, this method of determining exercise induced perfusion change is novel and theoretically justifiable. The force of the muscle contraction causes a rapid increase in the intramuscular pressure, expelling blood from capillaries causing a rapid decrease in the [tHb] signal. During relaxation, intramuscular pressure transiently returns to a level below the venous blood pressure and blood from the capillaries is pulled into and refills the veins until the next contraction. This phenomenon is known as the mechanical muscle pump effect (Tschakovsky et al. 1996). In this context, as stated above, the [tHb] signal reflects microvascular perfusion and O_2_ diffusing capacity. Accordingly, we propose that the range of the [tHb] signal over the contraction-relaxation cycle may be a function of the local muscle pump emptying and refilling capacity at a specific exercise intensity, vasodilatory response, and capillarity.

#### Muscle oxygen recovery capacity

Muscle oxygen recovery capacity (MORC) was assessed as the ${\mathrm{m}\dot{\mathrm{V}}\mathrm{O}}_{2}$ recovery rate constant (k) and reported as a time constant (tc) for mean values, which is equal to the inverse of k (tc = 1/k), detailed elsewhere (Ryan et al. 2012) (Table 2)⁠. Briefly, repeated AOs were done according to a set timing sequence following a brief bout of exercise measuring mV̇O_2_ over a 5-min period to assess recovery. The MORC tests were conducted with the participant in a seated position using knee-extension exercise as the exercise stimulus. The participant performed light, rapid and rhythmic 90˚ isotonic knee extension exercise for 10 s set to 16 N∙m with max extension set to 60˚ to obtain maximal VL activation and minimize recovery between contractions increasing mV̇O_2_ while maintaining muscle oxygen levels above 40% (Adami et al. 2017)⁠. Immediately after exercise, the participant was instructed to completely relax while their leg lifted to neutral position for the AOs, the first delivered 8 s after the last contraction. The AO sequence was piloted to optimize ${\mathrm{m}\dot{\mathrm{V}}\mathrm{O}}_{2}$ recovery kinetics with minimal disruption to blood flow in the current population and is as follows: AO 1–3: 5 s on/10 s off, AO 3–6: 6 s on/12 s off, AO 7–11: 7 s on/15 s off, AO 12–14: 10 s on/30 s off, AO 15: 30 s on then off. Estimates of mV̇O_2_ were calculated as the rate of change in the HbDif signal as described earlier. The individual mV̇O_2_ were plotted as a function of time and fit to a mono-exponential curve (Table 2). The recovery rate constant of mV̇O_2_ after exercise is proportional to the maximal oxidative capacity (Ryan et al. 2014)⁠ and was assessed the average of two consecutive tests.

#### Statistical analysis

The degree of test–retest reproducibility within NIRS devices was assessed using the intraclass correlation coefficient (ICC), standardized typical error (STE), and coefficient of variation as a percentage (CV) (Hopkins et al. 2009) were calculated within a published spreadsheet (Hopkins 2019) and defined in Table 2. Thresholds for ICC were: 0.20, low; 0.50, moderate; 0.75, high; 0.90, very high; and 0.99, nearly perfect (Hopkins et al. 2009), provided qualitative reference to the magnitude of reproducibility of the parameter. The STE gives the random error of the calibrated value standardized to the composite SD with the magnitude difference between NIRS devices interpreted according to the thresholds: 0.1, small; 0.3, moderate; 0.6, large; 1.0, very large; and 2.0, extremely large (Hopkins et al. 2009; Hopkins 2022). To estimate the probability of any substantial differences (i.e., Cohen *d* > 0.2) between the T2D and ND groups and between the NIRS technologies (devices), a mixed-model analysis of variance with adjustment for individual participant ATT was run using software (SAS 9.4, Cary, NC), where the random effect was the individual responses to NIRS device. The magnitude of the effect of ATT on the dependent was evaluated at 2SD.

Precision of the estimates was presented as 90% confidence limits, as favored by Rothman (2012). Decisions about magnitudes accounting for the precision (uncertainty) were based on two one-sided interval hypothesis tests as for non-inferiority, superiority, and equivalence testing (EMA 2000; Lakens 2018), where the hypothesis of a substantial difference between devices, either increase or decrease is rejected at the 5% level if the 90% confidence interval falls outside the threshold value for the opposing smallest standardized difference, which provides an alpha of 0.05 (5% maximum error rate of false rejection). Statistical differences are referenced relative to the threshold for smallest important difference, Cohen *d* = 0.2, with small sample-size adjustment according to Hedges (1981), where SD was the appropriate SD for the relative reference group (ND) or device (FD-NIRS): the basal SD for MVC comparisons, to FD-NIRS for the NIRS device comparisons, and the ND for between group comparisons. Accordingly, the probability that the alternative hypothesis was real was qualified into bins; for example, p > 0.95, was very good evidence for superiority (i.e., greater than), relative the smallest standardized difference ⁠(Hopkins et al. 2009; Hopkins 2022). Where the 90% confidence interval fell between the smallest effect thresholds for a positive and negative effect, the contrast was deemed equivalent ⁠(Hopkins 2022; Lakens 2018). False discovery rate of 10% rejection of non-inferiority on the opposite one-sided interval was used to adjust for the multiple familywise estimates (by group, by device, and by-group by-device contrasts for each outcome parameter).

## Results

Raw absolute means and standard deviations for all NIRS parameters for each NIRS device and group are shown in Table 2 presented within the labelled worksheets in Supplementary file 1. Reproducibility parameters are listed in Table 3.

**Table 3:** Reliability parameters for all NIRS outcome measures from frequency-domain (FD) and continuous-wave (CW) NIRS in men with (T2D) and without (ND) type-2 diabetes at rest and during knee-extension exercise at 5% and 15% of maximal voluntary contraction

Cohort	ND			T2D		
Device-reliability parameter	Rest	5%	15%	Rest	5%	15%
	*mBf*					
FD-ICC	0.39 (0.00, 0.73)	0.83 (0.62, 0.94)	0.91 (0.78, 0.97)	0.21 (0.00, 0.42)	0.91 (0.78, 0.97)	0.81 (0.57, 0.93)
- STE	0.81 (0.62, 1.19)	0.46 (0.35, 0.67)	0.35 (0.27, 0.51)	1.01 (0.62, 1.18)	0.34 (0.35, 0.67)	0.49 (0.27, 0.51)
- CV	60.3 (43.8, 99.3)	40.6 (30.0, 64.5)	36.5 (27.1, 57.6)	76.3 (54.7,129.9)	42.6 (31.4, 68.0)	54.7 (39.9, 89.2)
CW-ICC	0.78 (0.52, 0.91)	0.71 (0.41, 0.89)	0.84(0.63, 0.94)	0.77 (0.42, 0.89)	0.76 (0.49, 0.91)	0.83 (0.62, 0.93)
- STE	0.52 (0.40, 0.75)	0.58 (0.45, 0.85)	0.45 (0.35, 0.66)	0.57 (0.44, 0.84)	0.54 (0.42, 0.79)	0.49 (0.36, 0.67)
- CV	19.4 (14.6, 29.5)	56.2 (41.0, 92.0)	39.6 (29.3, 62.8)	25.3 (19.1, 41.7)	51.6 (37.8, 90.3)	51.5 (37.7, 83.6)
	*mV̇O*_*2*_					
FD-ICC	0.33 (0.00, 0.70)	0.86 (0.68, 0.96)	0.84 (0.63, 0.94)	0.74 (0.45, 0.90)	0.80 (0.55, 0.93)	0.92 (0.80, 0.97)
- STE	0.84 (0.65, 1.23)	0.42 (0.32, 0.61)	0.45 (0.35, 0.66)	0.56 (0.43, 0.82)	0.50 (0.39, 0.73)	0.32 (0.25, 0.47)
- CV	72.6 (52.2, 122.1)	30.4 (22.7, 47.4)	37.3 (27.7, 60.0)	28.6 (21.3, 44.4)	26.6 (19.9, 41.1)	27.3 (20.4, 42.3)
CW-ICC	0.39 (0.00, 0.73)	0.94 (0.85, 0.97)	0.96 (0.89, 0.94)	0.58 (0.21, 0.83)	0.98 (0.94, 0.99)	0.95 (0.87, 0.98)
- STE	0.81 (0.62, 1.18)	0.28 (0.22, 0.41)	0.24 (0.19, 0.36)	0.69 (0.53, 1.01)	0.18 (0.14, 0.26)	0.26 (0.20, 0.38)
- CV	53.4 (39.0, 86.9)	22.0 (16.5, 33.7)	18.0 (13.5, 27.3)	60.8 (44.2, 100.4)	18.8 (14.16, 28.6)	29.4 (21.9, 45.7)
	*[tHb]*					
FD-ICC	0.94 (0.86, 0.98)	0.96 (0.90, 0.98)	0.95 (0.88, 0.98)	0.97 (0.91, 0.99)	0.96 (0.92, 0.99)	0.96 (0.90, 0.99)
- STE	0.27 (0.21, 0.40)	0.23 (0.18, 0.33)	0.25 (0.19, 0.37)	0.21 (0.16, 0.31)	0.22 (0.17, 0.32)	0.23 (0.18, 0.33)
- CV	10.5 (8.0, 15.7)	9.4 (7.2, 14.1)	8.6 (6.6, 12.9)	7.1 (5.4, 10.5)	7.6 (5.8, 11.3)	07.5 (5.8, 11.2)
CW-ICC	0.96 (0.90, 0.98)	0.95 (0.87, 0.98)	0.95 (0.88, 0.98)	0.96 (0.88, 0.98)	0.96 (0.88, 0.98)	0.96 (0.88, 0.98)
- STE	0.22 (0.17, 0.32)	0.27 (0.20, 0.39)	0.25 (0.20, 0.37)	0.24 (0.19, 0.35)	0.23 (0.18, 0.34)	0.25 (0.19, 0.36)
- CV	3.5 (2.7, 5.1)	4.1 (3.1, 6.0)	4.0 (3.1, 6.0)	4.9 (3.7, 7.2)	5.2 (3.9, 7.6)	5.1 (3.9, 7.5)
	*SaO*_*2*_*/TSI*					
FD-ICC	0.75 (0.46, 0.90)	0.92 (0.79, 0.97)	0.90 (0.76, 0.97)	0.70 (0.91, 0.99)	0.87 (0.82, 0.91)	0.89 (0.84, 0.93)
- STE	0.55 (0.43, 0.81)	0.33 (0.26, 0.49)	0.36 (0.28, 0.38)	0.60 (0.46, 0.87)	0.40 (0.31, 0.59)	0.38 (0.29, 0.56)
- CV	5.0 (3.8, 7.4)	5.2 (4.0, 7.7)	8.8 (6.7, 13.1)	6.1 (4.6, 9.0)	5.6 (4.3, 8.3)	7.3 (5.6, 10.9)
CW-ICC	0.57 (0.21, 0.83)	0.73 (0.44, 0.90)	0.79 (0.55, 0.93)	0.79 (0.53, 0.92)	0.97 (0.91, 0.99)	0.97 (0.93, 0.99)
- STE	0.69 (0.53, 1.01)	0.56 (0.43, 0.82)	0.50 (0.39, 0.74)	0.51 (0.39, 0.74)	0.21 (0.16, 0.31)	0.19 (0.15, 0.28)
- CV	6.7 (5.1, 9.9)	4.5 (3.4, 6.6)	4.3 (3.3, 6.3)	2.5 (1.9, 3.7)	1.4 (1.1, 2.1)	1.9 (1.4, 2.7)
	*[tHb] Range*					
FD-ICC	–	0.90 (0.76, 0.97)	0.89 (0.74, 0.96)	–	0.88 (0.71, 0.95)	0.88 (0.72, 0.96)
- STE	–	0.36 (0.28, 0.53)	0.37 (0.29, 0.55)	–	0.39 (0.30, 0.57)	0.39 (0.31, 0.57)
- CV	–	29.5 (22.0, 45.9)	24.7 (18.5, 38.1)	–	40.6 (30.0, 64.5)	32.4 (24.1, 50.7)
CW-ICC	–	0.79 (0.53, 0.92)	0.72 (0.42, 0.90)	–	0.95 (0.87, 0.98)	0.96 (0.89, 0.96)
- STE	–	0.51 (0.39, 0.75)	0.58 (0.44, 0.84)	–	0.26 (0.20, 0.38)	0.25 (0.19, 0.44)
- CV	–	35.0 (26.0, 55.0)	42.9 (31.6, 68.5)	–	20.2 (15.2, 30.8)	19.7 (4.9, 30.1)

Data are the mean estimate and 90% confidence limits. Abbreviations: coefficient of variation in percent (CV), contraction-relaxation perfusion change ([tHb] range), intraclass correlation coefficient (ICC), oxygen consumption (${\mathrm{m}\dot{\mathrm{V}}\mathrm{O}}_{2}$), skeletal muscle blood flow (mBF), standardized typical error (STE), tissue oxygen saturation (SaO_2_%/TSI%), total hemoglobin concentration ([tHb])

### Skeletal muscle blood flow and oxygen consumption

**Fig. 2:**
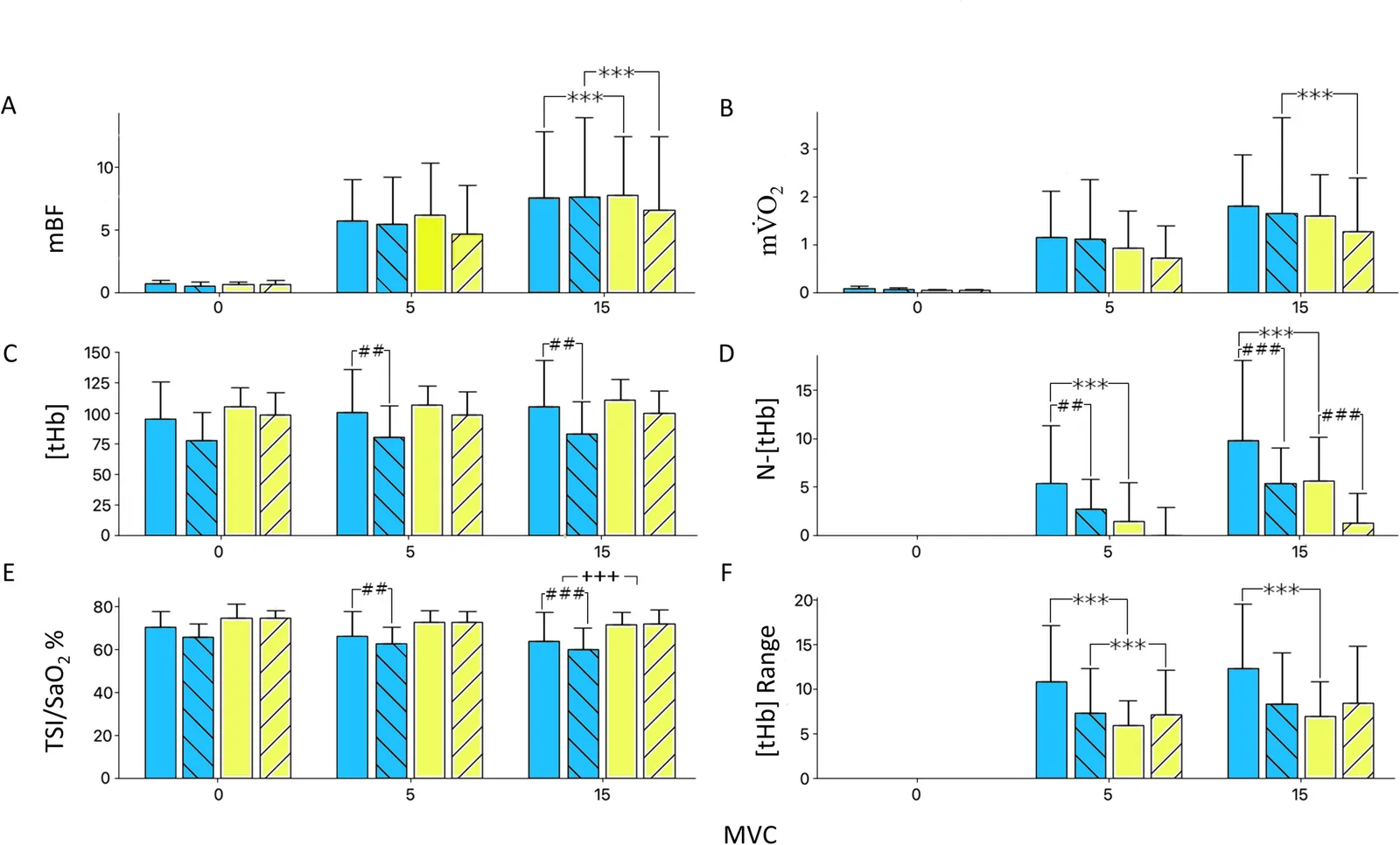
Quantitative agreement between hemodynamic and respiratory measures for non-diabetic (ND; solid) and type 2 diabetic (T2D; hashed) populations from frequency-domain (FD; blue) and continuous-wave (CW; yellow) NIRS at rest, 5% and 15% of maximal voluntary contraction (MVC). Data are mean values ± standard deviations. Mean values are staggered for clarity. The parameters are **A** skeletal muscle blood flow (mBF, mL·min^−1^·100 mL^−1^), **B** oxygen consumption (mV̇O_2_, O_2_(mL)∙min^-1^∙100g^-1^), **C** total hemoglobin concentration ([tHb] (μM)), **D** normalized increase in [tHb] from rest/baseline (N-[tHb] (μM)), **E** tissue oxygen saturation (SaO_2_%/TSI%) and **F** contraction-relaxation perfusion change ([tHb] range (μM)). The symbol # denotes substantial differences between T2D and ND groups, *symbol denotes substantial differences within-groups between-technology, and the + symbol denotes substantial differences between-technology between-groups. The strength of evidence for non-equivalency between devices or cohorts and probability bands is denoted: #/*, some evidence > 0.25 ##/**, good evidence > 0.75; ###/***, very good evidence > 0.95; ####/****, extremely good evidence > 0.995

For both test groups and NIRS devices the exercise mBF and mV̇O_2_ ICCs were high to very high (0.81–0.92), but ICCs at rest were low-moderate. Mean STEs were small-moderate across both devices and groups; STEs were substantially lower at rest with CW-NIRS for mBF, and at 15% MVC in ND and 5% MVC in T2D for ${\mathrm{m}\dot{\mathrm{V}}\mathrm{O}}_{2}$ (Table 3). For both groups and between NIRS devices, resting and exercise mBF and mV̇O_2_ values were not substantially different (Fig. 2). Substantial between-device within-group differences were seen at 15% MVC, while for mV̇O_2_ only T2Ds showed a substantial difference between devices.

Very good evidence was found for substantially differing measures between FD-NIRS and CW-NIRS in mBF at 15% MVC in both ND and T2D, and in mV̇O_2_ at 15% MVC for T2D (Fig. 2A, B). Finally, there was good to strong level evidence for an ATT influence on mBF in FD-NIRS (see Supplementary file 1, line items below “Estimate 2SD of ATT on mbf”) but not CW-NIRS in both ND and T2D groups, which also carried forward to between device estimates in T2D and between group estimates for FD.

The mBF/${\mathrm{m}\dot{\mathrm{V}}\mathrm{O}}_{2}$ and tHb/${\mathrm{m}\dot{\mathrm{V}}\mathrm{O}}_{2}$ ratios are in Table 4. For mBF/${\mathrm{m}\dot{\mathrm{V}}\mathrm{O}}_{2}$ the CW ratios were ~ 40% and ~ 60% higher when compared to FD in ND at rest and exercise, respectively. In contrast, in T2D, the same ratio was ~ 10% higher at rest, and ~ 7% higher only for 5% exercise while a ~ 33% decrease was seen in 15% exercise. For tHb/mV̇O_2_ the CW ratios were consistently higher than FD (~ 77% for ND at rest, ~ 30–40% for all other values) in both ND and T2D groups, and the T2D values were higher than ND values (~ 100% at rest for FD, ~ 50–65% for all other values).

**Table 4:** The muscle blood flow to oxygen consumption ratio (mBF/${\mathrm{m}\dot{\mathrm{V}}\mathrm{O}}_{2}$) and perfusion to oxygen consumption (tHb/${\mathrm{m}\dot{\mathrm{V}}\mathrm{O}}_{2}$) for rest, 5%, and 15% of knee extension maximal voluntary contraction in men with (T2D) and without (ND) type-2 diabetes

	ND			T2D		
	rest	5%	15%	rest	5%	15%
	*mBF/mV̇O*_*2*_					
FD	10.4 (5.2)	6.1 (2.3)	4.4 (2.0)	19.3 (26.3)	8.7 (6.0)	8.2 (7.0)
CW	14.1 (6.3)	9.5 (9.9)	7.0 (9.1)	21.4 (11.2)	9.3 (5.6)	6.2 (2.7)
	*tHb/mV̇O*_*2*_					
FD	1298 (347)	125 (70)	77 (49)	2567 (3218)	184 (159)	119 (105)
CW	2306 (623)	166 (91)	98 (77)	3308 (1496)	274 (214)	153 (118)

Data are mean (SD)

Abbreviations: *CW* continuous-wave NIRS, *FD* frequency-domain NIRS

#### Perfusion index

The ICC for perfusion index [tHb] was high to very high for both NIRS devices in both groups, except for moderate-large values at rest. STEs averaged small and there was no difference between devices or cohorts (Table 3). No differences between devices were seen in [tHb], however when [tHb] was normalized to resting measures (N-[tHb]) very good evidence for substantially higher perfusion was seen in FD-NIRS as compared to CW-NIRS in ND. In FD-NIRS there was good evidence for increased [tHb] in ND compared to T2D at 5 and 15% MVC. This same difference was seen in N-[tHb] at 5% MVC in ND, with very good evidence of increased N-[tHb] in both FD-NIRS and CW-NIRS at 15% MVC (Fig. 2C, D). There was very good evidence for substantial modification of [tHb] when measured by FD-NIRS (Supplementary file 1).

#### Tissue saturation of oxygen

The ICC value for tissue saturation of O_2_ at rest was moderate for both devices and groups, and exercise was high for both devices in both groups, except for CW-NIRS at 5% MVC in ND which was moderate. STEs averaged small-moderate, with a small relative increase with CW-NIRS in ND at 5% MVC, and small relative decreases in CW-NIRS in T2D at both 5% and 15% MVC (Table 3). The SaO_2_ value for FD-NIRS showed good and very good evidence for being substantially higher in ND compared to T2D at 5 and 15% MVC. Moreover, there was very good evidence for substantial differences between devices between groups at 15% MVC (Fig. 2E).

#### Contraction-relaxation perfusion change

The ICC values for contraction-relaxation perfusion change ([tHb] Range) between FD- and CW-NIRS were high for both groups, except CW-NIRS at 15% MVC in ND which was moderate. STEs were small-moderate and higher in CW-NIRS in ND at 15% MVC (Table 3). The contraction-relaxation perfusion change showed very good evidence for substantially higher change measured using FD-NIRS compared to CW-NIRS at 5% and 15% MVC in ND and at 5% MVC in T2D (Fig. 2F).

#### Muscle oxygen recovery capacity (MORC)

All effects between NIRS devices were statistically equivalent. The ${\mathrm{m}\dot{\mathrm{V}}\mathrm{O}}_{2}$ recovery time constant was 31 ± 91 s and 44 ± 140 s (rate constant: 0.0320 ± 0.0098 and 0.0225 ± 0.0035) for FD-NIRS in ND and T2D, respectively. The time constant was 29 ± 103 s and 37 ± 101 s (rate constant: 0.0340 ± 0.0074 and 0.0270 ± 0.0084) for CW-NIRS in ND and T2D, respectively. ICC values were high and low for FD-NIRS in ND and T2D, respectively, and low and moderate ICC values for CW-NIRS in ND and T2D, respectively.

## Discussion

The purpose of the current study was to compare and evaluate the quantitative agreement and reproducibility of hemodynamic and respiratory measures within the skeletal-muscle microvascular of healthy (ND) and insulin-resistant (T2D) individuals using CW-NIRS and FD-NIRS at rest and during dynamic exercise. Both devices demonstrated high reproducibility during exercise for the primary parameters of mBF, ${\mathrm{m}\dot{\mathrm{V}}\mathrm{O}}_{2}$, and perfusion ([tHb]) during exercise. However, at rest, statistics showed lower reproducibility for mBF and ${\mathrm{m}\dot{\mathrm{V}}\mathrm{O}}_{2}$; for mBF, STE was substantially lower in both groups (Table 3). Both devices had mean small-moderate STE (mean CV 19–76%), suggesting diagnostic resolution to at least the small-moderate standardized effects (Hedges’ g) with typical sample sizes. In addition to the findings at rest, CW-NIRS had lower STE of ${\mathrm{m}\dot{\mathrm{V}}\mathrm{O}}_{2}$ at 15% MVC and 5% MVC in ND and T2D, respectively. No differences in STE were seen for [tHb], but together with small-sized higher and lower contrast estimates for SaO_2_/TSI, the variability in within-subject within-device error does not support the study hypothesis of higher variability with CW-NIRS.

On the other hand, FD-NIRS detected quantitative group differences in absolute and normalized perfusion measures between ND and T2Ds at 5 and 15% MVC, while CW-NIRS only detected this difference in normalized perfusion at 15% MVC (Fig. 2C, D). These data are consistent with a precision advantage of FD-NIRS over CW-NIRS in sampling settings where heterogeneity in scattering within or across the sampled tissue is presented or expected, herein via increasing exercise intensity increased and different metabolic conditions within normal vs insulin-resistant tissue. Substantially lower perfusion and oxygenation may reflect slower skeletal-muscle microvascular kinetics (Bauer et al. 2007) and lower capillary density (Mårin et al. 1994) in T2D. These data imply better diagnostic potential of FD-NIRS to assess blood perfusion and tissue oxygen saturation in cohorts with impaired microvascular function. While the constant scattering assumptions of CW-NIRS appear to produce comparable quantitative outcomes to FD-NIRS for all parameters at rest and the low-intensity workload (5% MVC) for mBF and mV̇O_2_ measures, substantial between-device differences were also evident at 15% MVC and at a greater magnitude in T2D, which may also be caused by the physiological and technological scattering features.

When utilizing CW-NIRS, applying a single set of scattering coefficients across individuals does not account for scattering changes due to individual variation in muscle quality and make-up (Ferreira et al. 2006)⁠ and may be responsible for the different quantitative outcomes for [Hb]-derived parameters between devices. In previous work, calculating [Hb] parameters from literature-based scattering compared to measuring individual scattering-derived [Hb] showed substantial overestimations in [tHb] starting at 21% of peak incremental cycling exercise (Ferreira et al. 2006)⁠ and on the second bout of 6-min heavy leg cycling (Burnley et al. 2002)⁠. In FD-NIRS, assuming constant scattering compared with taking the instrument value significantly increased [ΔHb] over time under VO and AO, subsequently increasing the calculated values for mBF and ${\mathrm{m}\dot{\mathrm{V}}\mathrm{O}}_{2}$ (Hammer et al. 2019)⁠.

In alignment with skeletal-muscle metabolic workload, mBF and ${\mathrm{m}\dot{\mathrm{V}}\mathrm{O}}_{2}$ increased incrementally from rest to 15% MVC, with a pattern similar for both devices and groups (Fig. 2A, B). While more workloads would be required to establish a load-response equation, the patterns agree with our recent report in ND (Lucero et al. 2018)⁠ and with other established methodologies to measure mBF like thermodilution using the Fick principle during knee extension exercise (Blomstrand et al. 1997)⁠. With respect to the mBF/${\mathrm{m}\dot{\mathrm{V}}\mathrm{O}}_{2}$ ratio (Table 4), while not a main objective of this paper, an interesting finding was mBF/${\mathrm{m}\dot{\mathrm{V}}\mathrm{O}}_{2}$ ratios were higher in CW-NIRS compared to FD-NIRS in ND but were similar at rest and 5% exercise but noticeably lower in 15% exercise for T2D. We saw more consistency in tHb/${\mathrm{m}\dot{\mathrm{V}}\mathrm{O}}_{2}$ with higher ratios in CW-NIRS compared to FD-NIRS across both groups and higher ratios in T2D compared to ND across both devices. The mBF/${\mathrm{m}\dot{\mathrm{V}}\mathrm{O}}_{2}$ response is similar to values (~ 9–13) obtained using spatially resolved spectroscopy (SRS) CW-NIRS combined with indocyanine green dye flow during cycling exercise (Vogiatzis et al. 2015)⁠. To the best of our knowledge, however, our observations of higher microvascular perfusion to oxygen uptake at rest and during exercise in ND and T2D populations are novel, but consistent with hemodynamic pathophysiology in T2D (Bauer et al. 2007). Whilst marked disparity between experimental approaches and methodology used may provide the explanation, the higher mBF/${\mathrm{m}\dot{\mathrm{V}}\mathrm{O}}_{2}$ and tHb/${\mathrm{m}\dot{\mathrm{V}}\mathrm{O}}_{2}$ ratio measured with CW-NIRS at rest in both ND and T2D (Table 4) suggests different accuracy of measurement of these parameters within this device within the current leg model, at the least, of noteworthy magnitude of difference compared to our FD-NIRS and the two other methodologies.

Our findings demonstrate that while both FD-NIRS and CW-NIRS provide valuable insights into skeletal muscle microvascular and respiratory responses, critical quantitative distinctions emerge. Furthermore, we observed very good evidence that FD-NIRS provided substantially different measurements for mBF at 15% MVC in both ND and T2D, and for ${\mathrm{m}\dot{\mathrm{V}}\mathrm{O}}_{2}$ at 15% MVC in T2D. Similarly, FD-NIRS showed substantially higher N-[tHb] in the ND group and significantly greater contraction-relaxation perfusion change in certain conditions compared to CW-NIRS. This indicates a greater sensitivity or accuracy of FD-NIRS in capturing specific dynamic physiological changes. The mBF/${\mathrm{m}\dot{\mathrm{V}}\mathrm{O}}_{2}$ ratio also highlighted differences, with CW-NIRS yielding values almost double those of FD-NIRS at rest in the ND group and approximately 40% higher in the T2D group. These quantitative discrepancies emphasize that the choice of NIRS technology can influence NIRS outcomes and potentially the interpretation of the muscle hemodynamic responses, particularly when subtle differences or specific physiological thresholds are critical. For this reason, at least in part, CW-NIRS measures of perfusion are often presented as a percentage change from a physiological range (Ferrari et al. 2011)⁠.

### Limitations and future considerations

Each of the two NIRS devices were positioned on opposite legs to provide practical within-day within-participant control. The NIRS devices were randomized for leg dominance and starting leg, which will balance out any dominance related leg differences, but it is possible some variability was added to the between-device contrasts, although the within-device reproducibility would be unaffected.

The groups were not matched for age and BMI; however, age and higher BMI are characteristic of T2D (Fletcher et al. 2002). It should be acknowledged that any between-group differences may not be related solely to T2D, as age and BMI can play a significant contributory role also.

One cardiac cycle for mBF measures was determined using the pulsatile motion of the tHb signal⁠. It is also worth noting that since occlusion can only occur between contractions, the resulting [tHb] slope reflects post-contraction values and not dynamic exercise per se (Rådegran 1999)⁠. However, immediate post-exercise mBF increases in proportion to exercise intensity (Kagaya and Homma 1997)⁠ and reflects the mBF response to exercise (Quaresima et al. 2004)⁠.

Initially, we planned to assess both men and women. However, the T2D cohort was derived from a prior male-only exercise intervention. Consequently, we restricted our ND cohort to males, acknowledging that this limits our findings to this population. Future studies should include female participants to investigate potential sex differences in ATT, hemoglobin, and myoglobin concentrations, as previously reported (Craig et al. 2017), and to determine if these differences affect NIRS reproducibility and absolute values. Both NIRS devices are unable to distinguish between hemoglobin and myoglobin, and myoglobin has been said to contribute between 50 and 70% of NIRS derived [O_2_Hb] and [HHb] (Davis and Barstow 2013)⁠. We also recognize the potential influence of menstrual cycle phase on skeletal muscle hemodynamics (Limberg et al. 2010), which should be considered, or controlled for, in future female-inclusive studies. While expressing NIRS outcomes as a percentage of individual physiological range, obtained through vascular occlusion (5 min arterial occlusion ~ 250–300 mmHg) could normalize for varying ATT, during initial pilot trials we found this procedure uncomfortable for some participants, so excluded. Furthermore, although participants had similar skin tones, this was not documented. Future studies should record skin tone, as it has recently been identified as a potential confounding factor in NIRS measurements (Gómez et al. 2025).

In addition to potential sex differences, recent research confirms that ATT remains a primary confounding variable in NIRS-based assessments of skeletal muscle oxygenation and microvascular function, as it blunts signal sensitivity and reduces the detectable range of physiological changes (Landers-Ramos et al. 2024; Yáñez-Sepúlveda et al. 2023). We found good to strong evidence that ATT influenced mBF, [tHb] and during the higher MVC load mV̇O_2_ when measured in FD- but not CW-NIRS (Supplementary file 1), in both ND and T2D groups, suggesting FD- but not CW-NIRS is adjusting for ATT, at least to some extent, within data capture, which is consistent with our hypothesis. The current study did not focus specifically on the ATT confounder, but it is worthwhile examining recent reports for insight and future consideration in NIRS methods development. In a multiple regression analysis from data collected using CW-NIRS, ATT was the strongest predictor of leg mV̇O2 explaining 53.9% of the variance with age explaining 20.3% (van Beekvelt et al. 2025). In another study using CW-NIRS in healthy men and women, ATT was significantly correlated with StO_2_ slope 2, StO_2_ min, and StO_2_ range, and this was driven by female participants (Landers-Ramos et al. 2024). Interestingly, ATT (mean 2.3–2.4 mm) at the sample size were below values known to significantly reduce NIRS signal. These sex differences were eliminated when performing an ATT correction for StO_2_ slope 2, when using the T½ analysis, and when analyses were performed on a subset of males and females with similar ATT (Landers-Ramos et al. 2024). In clinical populations, ATT complicates the detection of microvascular impairments in conditions like chronic-obstructive pulmonary disease or peripheral artery disease (van Beekvelt et al. 2025). This limitation has prompted some researchers to utilize anatomical sites with minimal fat, such as the thenar eminence (base of thumb) (Hendrick et al. 2024) with obvious limitations or adopt advanced technical solutions like Dual-Slope FD-NIRS to isolate muscle- and adipose-tissue-specific data (Fernandez et al. 2023).

Additional research could further delineate the contribution of myoglobin to the NIRS signal at rest and during increasing exercise intensity, and the effect on calculated hemoglobin values for all NIRS measures. Due to limitations in CW-NIRS, it is helpful that researchers abide by recommendations and report parameters identified for optimizing validity and reproducibility, and to be able to compare results between studies (Ferrari et al. 2011)⁠. In the current study, FD-NIRS measures, a single scattering coefficient was calculated at rest and held constant throughout the experiment. Although it is known that the scattering coefficient changes during exercise (Koga et al. 2007)⁠, and because of the rapid occlusions (Hammer et al. 2019), it was assumed constant to isolate the effect of applying a single set of scattering correction factors to all participants as in CW-NIRS. Future research should investigate the effect of applying dynamic scattering on the same NIRS derived metrics and identify the effect of rapid occlusions on scattering and subsequent derived [Hb].

Meanwhile, it is important to note that the current data confirms the good utility of current protocols and of both CW-NIRS and FD-NIRS tools for studying skeletal muscle hemodynamic and respiratory response to dynamic exercise, but with respective limitations identified above. The current reproducibility data provides statistics to derive sample sizes to ensure sufficient power for the current tissue, rest, exercise model, equipment, lab conditions, and cohorts; any significant deviation will require new reproducibility data. Furthermore, within-subject research designs (e.g., randomized controlled trials with baseline adjustment; crossovers) control some of the technical challenges creating between-subject heterogeneity and measurement error and be used in preference when studying intervention or group differences.

Finally, with several important quantitative and sensitivity improvements with FD-NIRS evidenced, the practical implications differences should be considered. While FD-NIRS may offer greater sensitivity, the significantly higher instrument cost and greater operational complexity are important considerations. For many research and clinical applications where detecting general trends, relative changes, or gross physiological responses are sufficient, the ease of use and substantially lower cost of CW-NIRS devices may make them a more accessible and pragmatic option. Furthermore, for large-scale screening and educational purposes where accessibility and cost are paramount, CW-NIRS remains a highly valuable and viable option for skeletal muscle microvascular assessment. Therefore, the betterness of FD-NIRS in specific quantitative measures should be weighed against the practical advantages of CW-NIRS and the research question intricacies; furthermore, the latest generation of CW-NIRS devices may be more sensitive, but these were not tested in the current study. Researchers and clinicians should consider the specific research question, the desired level of sensitivity, and available resources when selecting NIRS technology. Nevertheless, for studies aiming to resolve subtle differences or requiring high absolute accuracy in populations with varying tissue characteristics, FD-NIRS may be the preferred tool.

## Conclusion

In summary, mBF, ${\mathrm{m}\dot{\mathrm{V}}\mathrm{O}}_{2}$, and perfusion derived from CW-NIRS and FD-NIRS at rest and up to 15% MVC of dynamic knee-extension exercise are reproducible and demonstrate an intensity-associated pattern of increase in agreement with previous research. FD-NIRS was more sensitive at detecting quantitative differences in absolute and normalized perfusion measures between T2D and ND. The constant scattering assumptions of CW-NIRS produced comparable quantitative outcomes to FD-NIRS for all parameters at rest, and for the low-intensity workload (5% MVC) for mBF and mV̇O_2_ measures, but substantial device differences were evident at 15% MVC and at a greater magnitude in T2D, suggesting non-agreement between devices increases as the magnitude of physiological scattering (e.g., tissue quality, ATT, skin color) and technological features also increase.

Overall, both NIRS devices proved to be reproducible and have potential as tools for non-invasive measurements of hemodynamic and oxygen uptake kinetics in rested and exercising skeletal muscle of healthy and T2D individuals. However, FD-NIRS was more sensitive to detect group differences in skeletal-muscle hemodynamic response to exercise, suggesting the NIRS technology was better for resolving differences in clinical or aged populations when compared to healthy young people. Nevertheless, the decision between FD-NIRS and CW-NIRS should be a pragmatic one, contingent on the specific research, budget availability, technical capability of users, or clinical application. Future research should continue to explore technical and methodological innovations, specifically around photon scattering, to enhance the accuracy of CW-NIRS and FD-NIRS instruments to improve experimental and clinical applications of this relatively low-cost non-invasive technology. 

## Supplementary Information

Below is the link to the electronic supplementary material.

Supplementary file1 (XLSX 60 KB)

## Abbreviations

AO: Rapid arterial occlusion
CV: Coefficient of variation
CW: Continuous wave
DPF: Differential path-length factor
FD: Frequency domain
ICC: Intraclass correlation coefficient
HbDif: Slope of the hemoglobin signal
HHb: Deoxygenated hemoglobin
MORC: Muscle oxygen recovery capacity
MVC: Maximal voluntary contraction
${\mathrm{m}\dot{\mathrm{V}}\mathrm{O}}_{2}$: Muscle volume of oxygen consumed, muscle oxygen uptake
mBF: Muscle blood flow
MVC: Maximum voluntary contraction
ND: Nondiabetic
NIRS: Near infrared spectroscopy
O_2_Hb: Oxygenated hemoglobin
SaO_2_: Arterial oxygen saturation
STE: Standardized typical error
T2D: Type-2 diabetic
tHb: Total hemoglobin
VL: *Vastus Lateralis*
VO: Rapid venous occlusion
